# Dietary Crude Protein Levels Alter Diarrhea Incidence, Immunity, and Intestinal Barrier Function of Huanjiang Mini-Pigs During Different Growth Stages

**DOI:** 10.3389/fimmu.2022.908753

**Published:** 2022-07-07

**Authors:** Yating Liu, Md. Abul Kalam Azad, Xichen Zhao, Qian Zhu, Xiangfeng Kong

**Affiliations:** ^1^ Hunan Provincial Key Laboratory of Animal Nutritional Physiology and Metabolic Process, Key Laboratory of Agro-ecological Processes in Subtropical Region, National Engineering Laboratory for Pollution Control and Waste Utilization in Livestock and Poultry Production, Institute of Subtropical Agriculture, Chinese Academy of Sciences, Changsha, China; ^2^ University of Chinese Academy of Sciences, Beijing, China

**Keywords:** diarrhea, dietary crude protein, huanjiang mini-pigs, immunity, intestinal barrier function

## Abstract

Huanjiang mini-pig is an indigenous pig breed in China; however, the optimal dietary crude protein (CP) levels for this pig breed during different growth stages has not been standardized yet. This study investigated the effects of different CP levels on diarrhea incidence, immunity, and intestinal barrier function in pigs. A total of 360 Huanjiang mini-pigs were assigned to three independent trials and fed the following CP diets: 5−10 kg stage, 14, 16, 18, 20, and 22%; 10−20 kg stage, 12, 14, 16, 18, and 20% and 20−30 kg stage, 10, 12, 14, 16, and 18%. In the 5−10 kg stage, the 22%; diet increased the plasma IL-1β, IL-6, IL-8, and TNF-α concentrations compared to the 14−20% diets and decreased IL-10 and TGF-β; however, these results were fluctuated in the later stages, including the decrease of IL-1β and IL-8 in the 20% group, TNF-α in the 18−20% groups, and the increase of IFN-γ in the 20% group at the 10−20 kg stage and the decrease of TNF-α in the 16% group at the 20−30 kg stage. The 20% diet increased the jejunal and ileal IL-10 concentration compared to the 14% diet at the 5−10 kg stage, as well as in the 16% diet compared to the 12% diet at the 10−20 kg stage. In addition, ileal IL-10 concentration was increased in the 16% diet compared to the 10, 12, and 18% diets at the 20−30 kg stage. Furthermore, the 18% diet at the 5−10 kg stage and the 16% diet at the 10−20 kg stage decreased jejunal IL-6 expression, whereas the 20% diet increased the TNF-α and IFN-γ at the 5−10 kg stage. The 20% diet increased the Claudin, Occludin, ZO-1, ZO-2, Mucin-1, and Mucin-20 expressions at the 5−10 kg stage, as well as TLR-2, TLR-4, and NF-κB in the 22 and 20% diets at the 5−10 and 10−20 kg stages, respectively. Collectively, these findings suggest optimal dietary CP levels of 16, 14, and 12% for Huanjiang mini-pigs during the 5−10, 10−20, and 20−30 kg growth stages, respectively; and provide the guiding significance of dietary CP levels for Huanjiang mini-pigs during different growth stages.

## Introduction

Protein is a fundamental and essential nutrient for normal growth and development of animals, and dietary protein is one of the main energy sources for swine. China has a high demand for soybeans, half of which are imported, and the price of soybeans has been rising (1). Increasing feed costs entail a reduction in the proportion of protein in the diet. However, environmental pollution caused by nitrogen emissions also necessitates new requirements for the sustainable development of animal husbandry. Excessive intake of dietary crude protein (CP) can damage the intestinal barrier function of piglets, and a large amount of dietary CP consumption by growing-finishing pigs results in a large amount of nitrogen emission. Recent studies have shown that nitrogen excretion is reduced by approximately 8.0% for each 10 g/kg reduction in dietary CP (2). Therefore, it is important to optimize dietary CP levels to reduce costs of protein feedstuff, improve body health, and reduce environmental pollution by excreta.

Huanjiang mini-pigs are a famous local miniature pig breed in China that have excellent meat quality, genetic stability, tolerance to roughage feeding, and strong disease resistance (3). However, their low growth and development, traditional free-range farming, high mortality, and low extensive farming efficiency have severely hindered their large-scale production. Most studies on this pig breed have focused on meat quality (4) and reproductive performance (3), which does not take into account the standards for nutrient, and especially protein, requirements. Therefore, it is of great significance to establish the nutritional requirements of Huanjiang mini-pigs at different growth stages.

The small intestine is the main site for protein digestion and absorption and is also an important immune organ. Therefore, the maintenance of intestinal health could influence the growth performance (5) and immune status (6) of pigs. Lan et al. (7) suggested that high protein (HP) diets led to lower ileal and colonic indices in rats related to the mucosal immune response. Yin et al. (8) also indicated that dietary HP may cause diarrhea in piglets by inducing intestinal inflammation through the activation of the NF-κB signaling pathway. Our previous study found that increasing levels of dietary CP quadratically decreased the feed to body weight gain (F/G) during the 5−10 kg growth stage, while the average daily gain (ADG) during the 10−20 kg growth stage and average daily feed intake (ADFI) and ADG during the 20−30 kg growth stage were quadratically increased (9). Based on the above-mentioned findings, we hypothesized that the positive roles of optimal dietary CP levels in growth performance might be related to their effects on the immune function and gut health of pigs. Based on the National Research Council (NRC, 2012) (10) recommended dietary CP levels, we designed diets with an increase of two percentage points and a decrease of two to six percentage points. Therefore, the present study was conducted to investigate the effects of dietary CP levels on intestinal barrier and immune function of Huanjiang mini-pigs. This study will serve to provide a basis for optimizing the dietary CP formula of Huanjiang mini-pigs.

## Materials and Methods

### Animals, Management, and Diet

**Figure 1:**
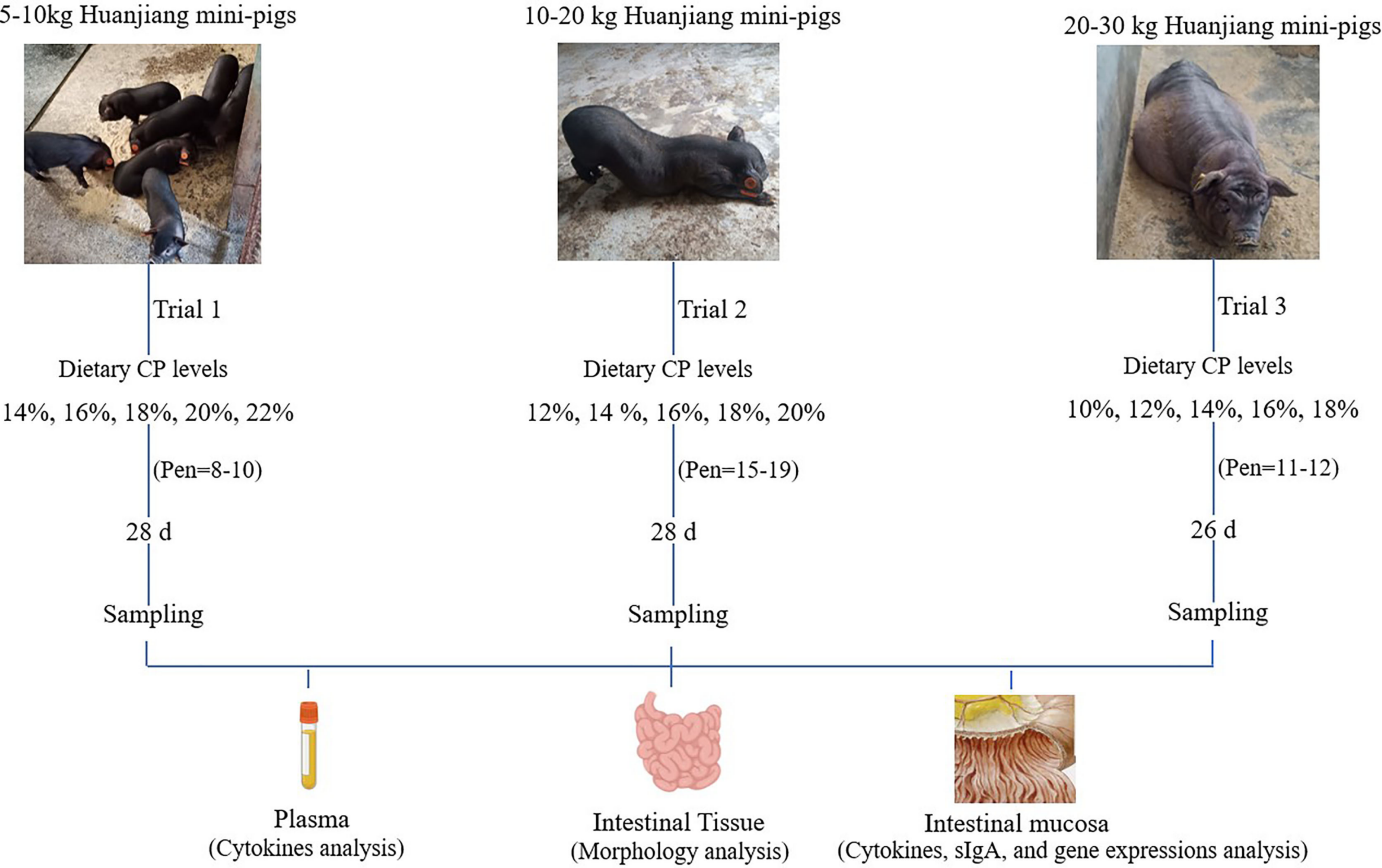
Schematic presentation of the experimental design of dietary crude protein (CP) levels on diarrhea incidence, immunity, and intestinal barrier function of Huanjiang mini-pigs during different growth stages.

This study was conducted at the Huanjiang Observation and Research Station for Karst Ecosystems (E108.41265°C, N 25.09043°C) in Huanjiang, Guangxi, China. A total of 360 Huanjiang mini-pigs (male: female, 1:1) were used to conduct three independent trials based on different growth stages [5−10, 10−20, and 20−30 kg body weight (BW)], which lasted for 28, 28, and 26 days, respectively. In the 5−10 kg growth stage, 220 pigs (5.32 ± 0.46 kg BW) were randomly divided into five groups (each group had 8−10 pens and five pigs per pen) and fed with 14, 16, 18, 20, and 22% CP diets. In the 10−20 kg growth stage, 84 pigs (11.27 ± 1.43 kg BW) were randomly divided into five groups (each group had 15−19 pens and one pig per pen) and fed with 12, 14, 16, 18, and 20% CP diets. In the 20−30 kg growth stage, 56 pigs (18.80 ± 2.21 kg BW) were randomly assigned to five groups (each group had 11−12 pens and one pig per pen) and fed with 10, 12, 14, 16, and 18% CP diets (**Figure 1**).

The pigs were fed three times daily (08:00, 14:00, and 20:30) and had *ad libitum* access to water and diet at all times. Dietary CP levels were designed according to both the National Research Council (NRC, 2012) (10) recommended requirements, and the Chinese nutrient requirements for swine in China (NY/T65-2004) (11) (**Supplementary Tables S1–S3**). The premixes were formulated to be isoenergetic using recommended values of individual ingredients according to NRC (2012) (10).

### Sample Collection

At the end of each trial, one pig with an average BW was selected from each pen for the collection of samples after electrical stunning (120 V, 200 Hz). Blood samples (10 mL per pig) were collected from the precaval vein into heparin-treated tubes, centrifuged at 3500 × *g* for 10 min at 4°C to obtain plasma, and immediately stored at −20°C for cytokine analysis. The jejunum (10 cm below the flexure of the duodenum-jejunum) and ileum (10 cm above the ileocecal junction) tissues were sampled (1−2 cm length), washed with cold phosphate-buffered saline (PBS), and then fixed with 4% paraformaldehyde for morphological analysis. The intestinal mucosa was scraped, frozen in liquid nitrogen, and stored at −80^ο^C to analyze cytokines, secretory immunoglobulin A (sIgA), and gene expression related to immunity, tight junction protein, and mucin.

### Diarrhea Evaluation

The degree of diarrhea in Huanjiang mini-pigs in each group was assessed daily by Hart and Dobb’s method (12). Scores were 0, normal, firm feces; 1, mild diarrhea; 2, moderate diarrhea, definitely unformed, moderately fluid feces; and 3, severe diarrhea, very watery, and frothy diarrhea. The diarrhea rate and index were calculated as previously described (13). Briefly, the following equations were used:


Diarrhea rate(%)=total diarrhea times/(total number of pigs)×trial days×100.

Diarrhea index=(times of mild diarrhea×1+times of moderate diarrhea×2+times of severe diarrhea×3)/total number of diarrhea incidence.

### Intestinal Histomorphology Observation

The fixed jejunum and ileum segments were dehydrated using a gradient of alcohol and xylene and then embedded in paraffin. Tissues were cut into 5−6 μm thick sections and mounted on a glass slide. Hematoxylin and eosin (H&E) staining was performed as previously described (14). Villus height (VH) and crypt depth (CD) were observed using a light microscope (Olympus Bx51, Japan) at 40 × magnification and measured using Image-Pro Plus software (version 6.0) as described previously (15). In addition, the VH-to-CD ratio was calculated.

### Plasma Cytokines Analysis

The plasma concentrations of interleukin (IL)-1β, -6, -8, -10, tumor necrosis factor (TNF)-α, transforming growth factor (TGF)-β, and interferon (IFN)-γ were determined using commercially available porcine-specific ELISA kits from Jiangsu Meimian Industrial Co., Ltd. (Meimian, Yancheng, China) according to the manufacturer’s instructions. Absorbance values were measured using a Multiscan Spectuum Spectrophotometer (Infinite M200Pro, Tecan, Switzerland).

### Determination of Mucosal sIgA and Cytokines

According to the protocol described by Wang et al. (16), samples (~0.1000 g) of the jejunal and ileal mucosa were mixed with PBS (1:9, w:v) and homogenized, and the supernatants were obtained by centrifugation at 2000 × *g* for 10 min at 4°C. The concentrations of sIgA, IL-6, IL-10, IL-17, IFN-γ, TNF-α, and TGF-β were measured using commercially available porcine-specific ELISA kits (Jiangsu Meimian Industrial Co., Ltd.). A Pierce BCA Protein Assay Kit (Thermo Scientific, Shanghai, China) was used to quantify the total protein concentration in the jejunal and ileal mucosa. The concentration of each index was normalized to the total protein in each sample. Absorbance was measured using a Multiscan Spectrum Spectrophotometer (Infinite M200Pro, Tecan).

### Small Intestinal Mucosa RNA Extraction and Gene Expression Analysis

The jejunal and ileal mucosa samples were ground into powder with liquid nitrogen, and total RNA was isolated using TRIZOL reagent (Magen, Guangzhou, China). The extracted RNA was reverse-transcribed into cDNA using a PrimeScript RT reagent kit with gDNA Eraser (TaKaRa Biotechnology Co., Ltd., Dalian, China). Real-time reverse transcription polymerase chain reaction (RT-PCR) assays were conducted using the SYBR^®^ Premix Ex Taq™ Kit (TaKaRa Biotechnology Co., Ltd.) on a LightCycler^®^ 480 II Real-Time PCR System (Roche, Basel, Switzerland) (16). The RT-PCR conditions were as follows: initial denaturation at 95°C for 30 s, followed by 40 cycles of denaturation at 95°C for 5 s, annealing at 60°C for 30 s, and a final extension at 72°C for 30 s. Primers were designed and synthesized by Sangon Biotech (Shanghai) Co., Ltd., (Shanghai, China) (**Supplementary Table S4**). Target gene expression was normalized against the housekeeping gene β-actin and calculated using the 2^-ΔΔCt^ method (17).

### Statistical Analysis

The data were analyzed by one-way ANOVA, and the comparative analysis was performed using Tukey’s *post-hoc* test (SPSS 22.0; SPSS, Inc., Chicago, IL, USA). Individual piglets were used as the experimental unit. All data are presented as means ± standard error (SE). Differences between significant means were considered to be statistically different when *P* < 0.05, and 0.05 ≤ *P* < 0.10 were considered a trend. GraphPad Prism 8.0 (San Diego, CA, USA) was used to plot images.

## Results

### Diarrhea Rate and Index of Huanjiang Mini-Pigs

The effects of dietary CP levels on the diarrhea rate and index of pigs are presented in **Table 1**. In the 5−10 kg growth stage, the 20% CP diet increased (*P* < 0.05) the diarrhea index but not the diarrhea rate (*P* > 0.05) of pigs compared to pigs in the 14, 16, and 22% CP groups. In the 10−20 kg growth stage, the diarrhea rate but not the diarrhea index of pigs increased (*P* < 0.05) with the increase in dietary CP levels, and the 18 and 20% CP diets increased (*P* < 0.05) the diarrhea rate compared to pigs in the 12, 14, and 16% CP groups. In the 20−30 kg growth stage, the diarrhea rate and diarrhea index were increased (*P* < 0.05) with increased dietary CP levels. The 18% CP diet increased (*P* < 0.05) the diarrhea rate compared to the pigs in the other four CP groups, whereas the 16 and 18% CP diets increased (*P* < 0.05) the diarrhea index compared to the pigs in the 10, 12, and 14% CP groups.

**Table 1:** Effects of dietary crude protein (CP) levels on the diarrhea incidence of Huanjiang mini-pigs during different growth stages.

Items	Dietary CP levels (%)				
5−10 kg growth stage	14	16	18	20	22
Diarrhea rate (%)	13.45 ± 1.62	15.80 ± 2.35	14.54 ± 3.09	18.18 ± 2.92	18.96 ± 3.35
Diarrhea index	1.80 ± 0.16^b^	2.01 ± 0.04^b^	2.13 ± 0.03^ab^	2.18 ± 0.05^a^	1.80 ± 0.16^b^
10−20 kg growth stage	12	14	16	18	20
Diarrhea rate (%)	13.36 ± 2.33^b^	24.63 ± 4.16^b^	28.31 ± 7.54^b^	49.55 ± 10.15^a^	46.39 ± 6.63^a^
Diarrhea index	1.10 ± 0.23	1.07 ± 0.16	1.08 ± 0.17	1.05 ± 0.20	1.14 ± 0.07
20−30 kg growth stage	10	12	14	16	18
Diarrhea rate (%)	15.61 ± 3.62^b^	10.50 ± 3.05^b^	12.87 ± 4.31^b^	20.79 ± 3.90^b^	34.27 ± 3.78^a^
Diarrhea index	1.12 ± 0.10^ab^	0.92 ± 0.17^b^	0.69 ± 0.17^b^	1.46 ± 0.16^a^	1.38 ± 0.13^a^

Data are presented as means ± SE (n = 6−8 per group). Different superscript letters within the same row indicate significant differences among the five groups (P < 0.05).

### Small Intestinal Morphology of Huanjiang Mini-Pigs

**Figure 2:**
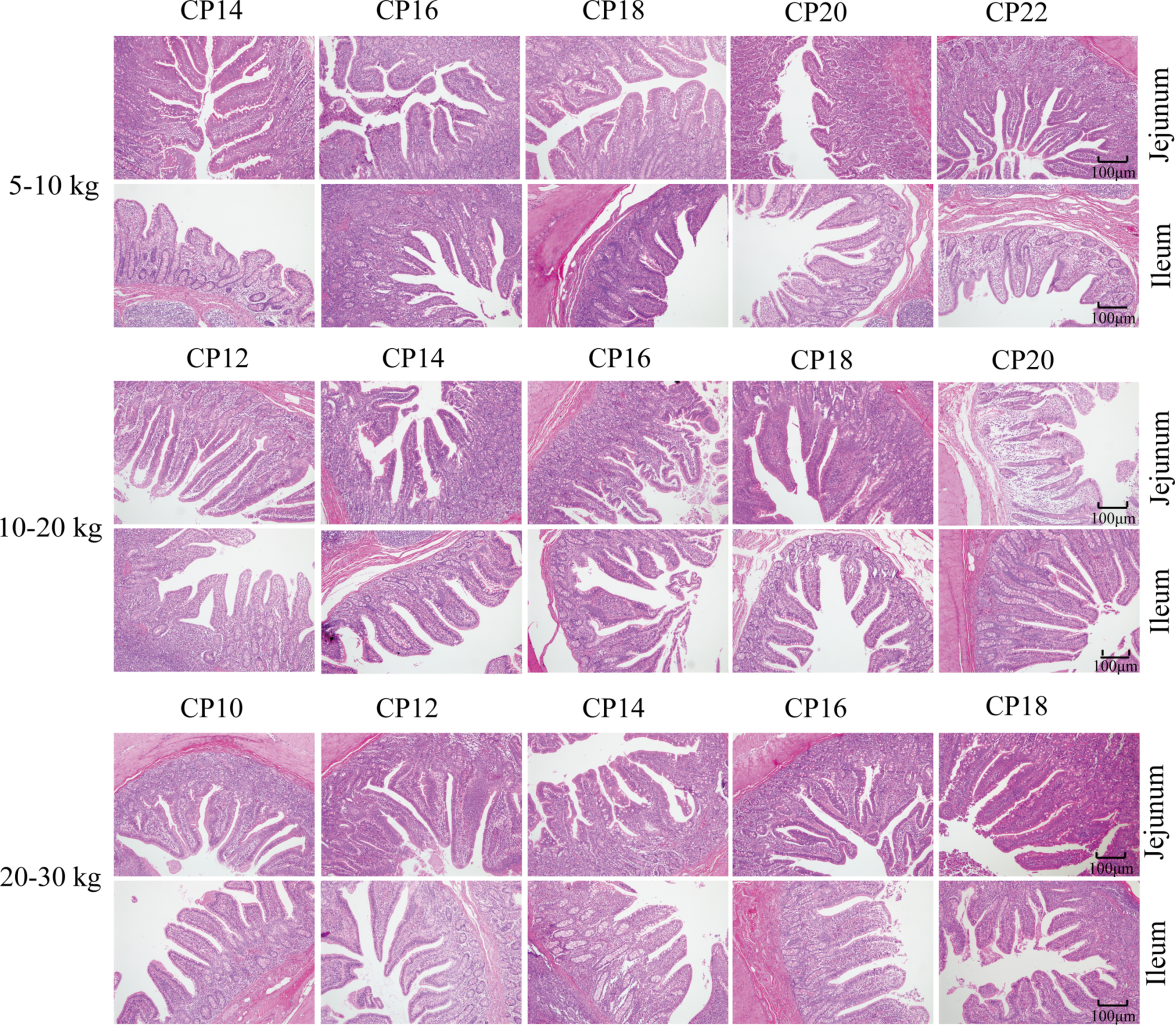
Effects of dietary crude protein (CP) levels on the small intestinal morphology of Huanjiang mini-pigs during different growth stages (*n* = 6−8 per group). H&E staining, 100 ×. CP, crude protein.

The effects of dietary CP levels on small intestinal morphology of pigs are presented in **Table 2** and **Figure 2**. In the 5−10 kg growth stage, the 18% CP diet increased (*P* < 0.05) VH in the jejunum compared to the other four CP groups. However, dietary CP levels did not affect VH, CD, or VH to CD ratio in the ileum (*P* > 0.05). In the 10−20 and 20−30 kg growth stages, there was no significant difference (*P* > 0.05) in VH, CD, and VH to CD ratio in the jejunum and ileum of piglets among the five CP groups (**Table 2**). Moreover, there were no obvious changes in the jejunum and ileum of pigs in the different CP groups (**Figure 2**).

**Table 2:** Effects of dietary crude protein (CP) levels on the small intestinal morphology of Huanjiang mini-pigs during different growth stages.

Items (μm)	Dietary CP levels (%)				
5−10 kg growth stage	14	16	18	20	22
Jejunum					
Villus height	327.30 ± 64.62^b^	316.17 ± 36.19^b^	399.50 ± 64.43^a^	320.60 ± 13.34^b^	323.60 ± 24.92^b^
Crypt depth	167.19 ± 12.23	172.04 ± 12.69	176.22 ± 11.14	148.29 ± 10.60	148.85 ± 7.35
VH/CD	2.42 ± 0.22	2.10 ± 0.17	2.33 ± 0.16	2.49 ± 0.09	2.33 ± 0.18
Ileum					
Villus height	289.80 ± 25.24	304.90 ± 19.97	290.30 ± 23.46	331.70 ± 54.73	292.60 ± 15.95
Crypt depth	163.34 ± 7.41	158.23 ± 8.34	159.54 ± 5.78	146.03 ± 7.22	149.22 ± 6.80
VH/CD	1.93 ± 0.12	2.08 ± 0.09	1.93 ± 0.13	2.54 ± 0.37	2.11 ± 0.17
10−20 kg growth stage	12	14	16	18	20
Jejunum					
Villus height	402.60 ± 24.66	416.15 ± 33.89	423.74 ± 25.36	399.11 ± 14.04	383.50 ± 25.90
Crypt depth	193.41 ± 15.74	172.40 ± 15.14	173.88 ± 12.26	174.31 ± 7.15	165.70 ± 8.40
VH/CD	2.11 ± 0.12	2.44 ± 0.13	2.46 ± 0.09	2.31 ± 0.09	2.33 ± 0.16
Ileum					
Villus height	341.38 ± 25.97	344.33 ± 23.22	337.83 ± 23.72	335.73 ± 6.57	355.62 ± 10.53
Crypt depth	173.44 ± 8.15	161.90 ± 6.95	173.99 ± 7.87	164.51 ± 6.21	171.51 ± 11.49
VH/CD	1.97 ± 0.12	2.15 ± 0.17	1.93 ± 0.08	2.07 ± 0.10	2.10 ± 0.10
20−30 kg growth stage	10	12	14	16	18
Jejunum					
Villus height	433.29 ± 37.10	434.04 ± 28.71	423.74 ± 25.36	399.11 ± 14.04	383.50 ± 25.90
Crypt depth	198.88 ± 14.38	162.48 ± 13.30	173.88 ± 12.26	174.31 ± 7.15	165.70 ± 8.40
VH/CD	2.19 ± 0.13	2.76 ± 0.24	2.46 ± 0.09	2.31 ± 0.09	2.33 ± 0.16
Ileum					
Villus height	341.96 ± 24.96	347.49 ± 14.62	343.58 ± 22.37	360.68 ± 31.02	330.28 ± 18.56
Crypt depth	166.12 ± 6.46	166.42 ± 4.17	159.95 ± 7.35	161.62 ± 5.48	167.67 ± 9.13
VH/CD	2.06 ± 0.12	2.09 ± 0.08	2.15 ± 0.13	2.23 ± 0.16	1.98 ± 0.10

Data are presented as means ± SE (n = 6−8 per group). Different superscript letters within the same row indicate significant differences among the five groups (P < 0.05). VH/CD, villus height to crypt depth.

### Plasma Cytokine Concentrations of Huanjiang Mini-Pigs

**Figure 3:**
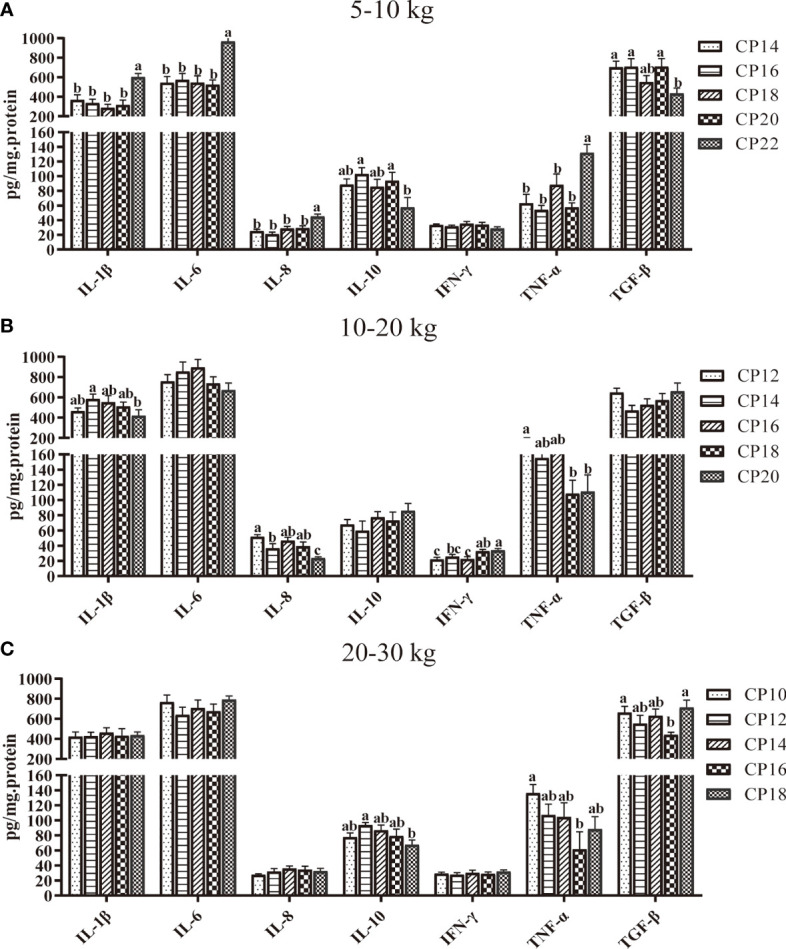
Effects of dietary crude protein (CP) levels on the plasma cytokine concentrations of Huanjiang mini-pigs during 5−10 kg **(A)**, 10−20 kg **(B)**, and 20−30 kg **(C)** growth stages. Data are presented as means ± SE (*n* = 6−8 per group). Different superscript letters indicate significant differences among the five groups (*P* < 0.05). IL, interleukin; IFN, interferon; TNF-α, tumor necrosis factor-alpha; TGF-β, transforming growth factor-beta.

The effects of dietary CP on plasma cytokine concentrations in pigs are shown in **Figure 3**. In the 5−10 kg growth stage, the 22% CP diet decreased (*P* < 0.05) the plasma IL-10 and TGF-β concentrations compared to the 16 and 20% CP groups but increased (*P* < 0.05) the plasma IL-1β, IL-6, IL-8, and TNF-α concentrations compared to the other four CP groups (**Figure 3A**). In the 10−20 kg growth stage, the 14 and 20% CP diets decreased the plasma IL-8 concentration, and the 18 and 20% CP diets decreased plasma TNF-α concentration but increased IFN-γ concentration when compared with the 12% CP group (*P* < 0.05). Moreover, the 20% CP diet decreased (*P* < 0.05) the plasma IL-1β and IFN-γ concentrations compared to the 14% CP group and IL-8 concentration compared to the 14, 16, and 18% CP groups, whereas the plasma IFN-γ concentration increased (*P* < 0.05) compared to the 16% CP group (**Figure 3B**). In the 20−30 kg growth stage, the 16% CP diet decreased (*P* < 0.05) the plasma TNF-α and TGF-β concentrations compared to the 10% CP group. The 18% CP diet decreased (*P* < 0.05) the plasma IL-10 concentration compared to the 12% CP group, whereas it increased (*P* < 0.05) the TGF-β concentration compared to the 16% CP group (**Figure 3C**).

### Secretory Immunoglobulin A Concentration in the Small Intestinal Mucosa of Huanjiang Mini-Pigs

**Figure 4:**
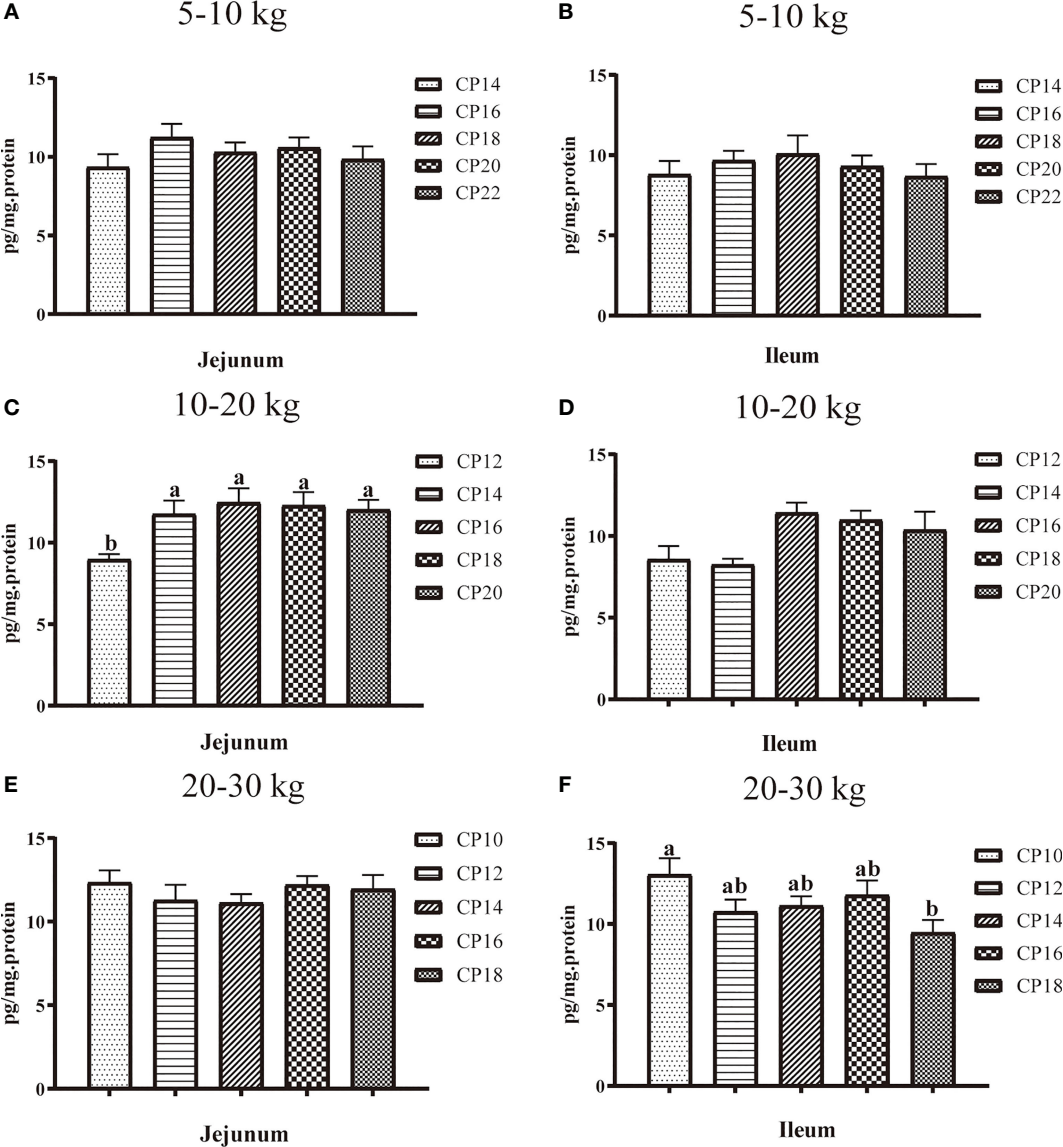
Effects of dietary crude protein (CP) levels on the small intestinal mucosa sIgA concentration of Huanjiang mini-pigs during 5−10 kg (**A**, jejunum; **B**, ileum), 10−20 kg (**C**, jejunum; **D**, ileum), and 20−30 kg (**E**, jejunum; **F**, ileum) growth stages. Data are presented as means ± SE (*n* = 6−8 per group). Different superscript letters indicate significant differences among the five groups (*P* < 0.05). sIgA, secretory immunoglobulin A.

The effects of dietary CP levels on jejunal and ileal sIgA concentrations in pigs are shown in **Figure 4**. In the 5−10 kg growth stage (**Figure 4A, B**), dietary CP levels did not affect (*P* > 0.05) the sIgA concentration in the jejunum and ileum of pigs. In the 10−20 kg growth stage (**Figure 4C, D**), the 12% CP diet decreased (*P* < 0.05) the jejunal sIgA concentration compared to the other four CP groups. In the 20−30 kg growth stage (**Figure 4E, F**), the 18% CP diet decreased (*P* < 0.05) the ileal sIgA concentration compared to the 10% CP group.

### Cytokine Concentrations in the Small Intestinal Mucosa of Huanjiang Mini-Pigs

**Figure 5:**
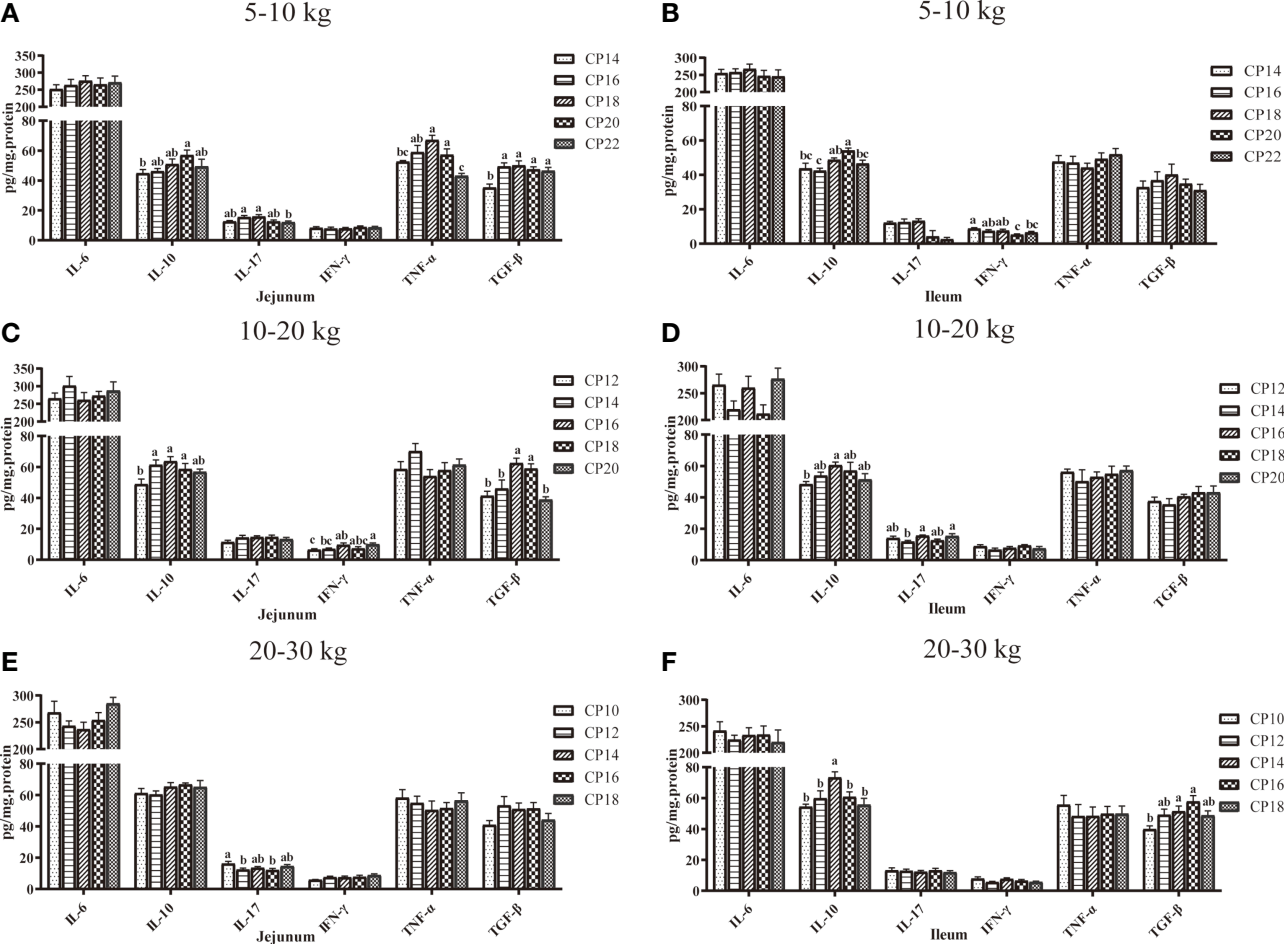
Effects of dietary crude protein (CP) levels on cytokine concentrations in the small intestinal mucosa of Huanjiang mini-pigs during 5−10 kg (**A**, jejunum; **B**, ileum), 10−20 kg (**C**, jejunum; **D**, ileum), and 20−30 kg (**E**, jejunum; **F**, ileum) growth stages. Data are presented as means ± SE (*n* = 6−8 per group). Different superscript letters indicate significant differences among the five groups (*P* < 0.05). IL, interleukin; IFN, interferon; TNF-α, tumor necrosis factor-alpha; TGF-β, transforming growth factor-beta.

The effects of dietary CP levels on cytokine concentrations in the small intestinal mucosa of pigs are presented in **Figure 5**. In the 5−10 kg growth stage (**Figures 5A, B**), the TNF-α concentration in the 18% CP group, IL-10 and TNF-α concentrations in the 20% CP group, and TGF-β concentration in the 16-22% CP groups were increased (*P* < 0.05) in the jejunal mucosa of pigs compared to the 14% CP group. In addition, the 22% CP diet decreased (*P* < 0.05) jejunal IL-17 and TNF-α concentrations compared to the 16 and 18% CP groups, whereas the 22% CP diet decreased the TNF-α concentration compared to the 20% CP group. In the ileal mucosa, the 18% CP diet increased (*P* < 0.05) the IL-10 concentration compared to the 16% CP group, whereas the 20% CP diet increased (*P* < 0.05) the IL-10 concentration compared to the 14 and 16% CP groups and decreased (*P* < 0.05) the IFN-γ concentration compared to the 14, 16, and 18% CP groups. Moreover, the 22% CP diet decreased (*P* < 0.05) the IFN-γ concentration compared to the 14% CP group and IL-10 concentration compared to the 20% CP group. In the 10−20 kg growth stage (**Figures 5C, D**), in the jejunal mucosa, the IFN-γ concentration in the 16% CP group and IL-10 concentration in the 14−18% CP groups were increased (*P* < 0.05) compared to the 12% CP group, whereas the IFN-γ concentration was increased (*P* < 0.05) in the 20% CP group compared to the 12 and 14 CP groups. In addition, the 16 and 18% CP diets increased (*P* < 0.05) the TGF-β concentration compared to the other CP groups. In the ileal mucosa, the 16% CP diet increased the IL-10 concentration compared to the 12 and 14% CP groups, whereas the 20% CP diet increased the IL-17 concentration compared to the 14% CP group (*P* < 0.05). In the 20−30 kg growth stage (**Figures 5E, F**), the 12 and 14% CP diets decreased (*P* < 0.05) the jejunal IL-17 concentration compared to the 10% CP group. In addition, the 14 and 16% CP diets increased the ileal TGF-β concentration compared to the 10% CP group, while the 14% CP diet increased the IL-10 concentration compared to the other CP groups (*P* < 0.05).

### Immunity-Related Gene Expression in the Small Intestinal Mucosa of Huanjiang Mini-Pigs

**Figure 6:**
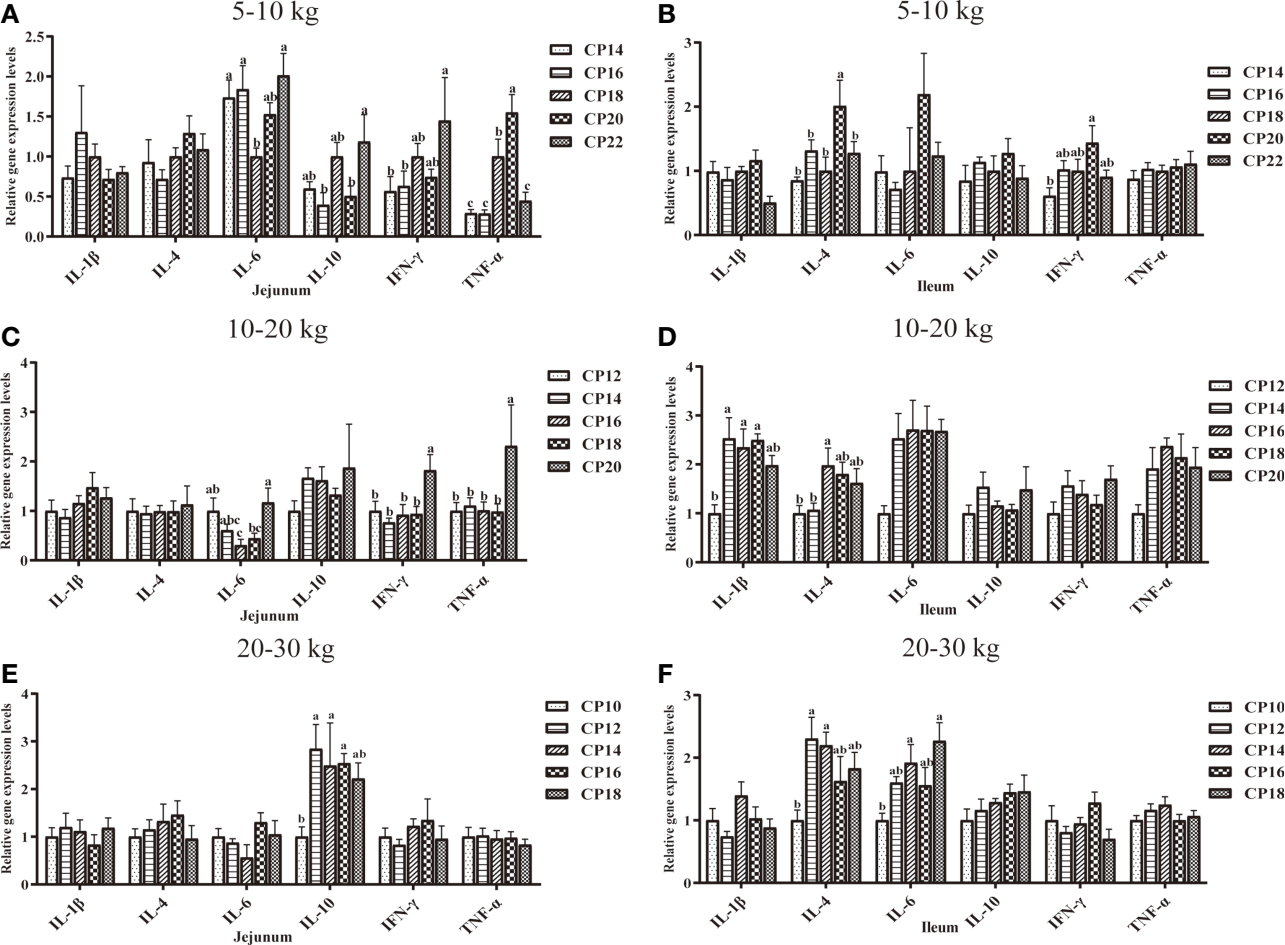
Effects of dietary crude protein (CP) levels on the immunity-related gene expression in the small intestinal mucosa of Huanjiang mini-pigs during 5−10 kg (**A**, jejunum; **B**, ileum), 10−20 kg (**C**, jejunum; **D**, ileum), and 20−30 kg (**E**, jejunum; **F**, ileum) growth stages. Data are presented as means ± SE (*n* = 6−8 per group). Different superscript letters indicate significant differences among the five groups (*P* < 0.05). IL, interleukin; IFN, interferon; TNF-α, tumor necrosis factor-alpha. .

**Figure 7:**
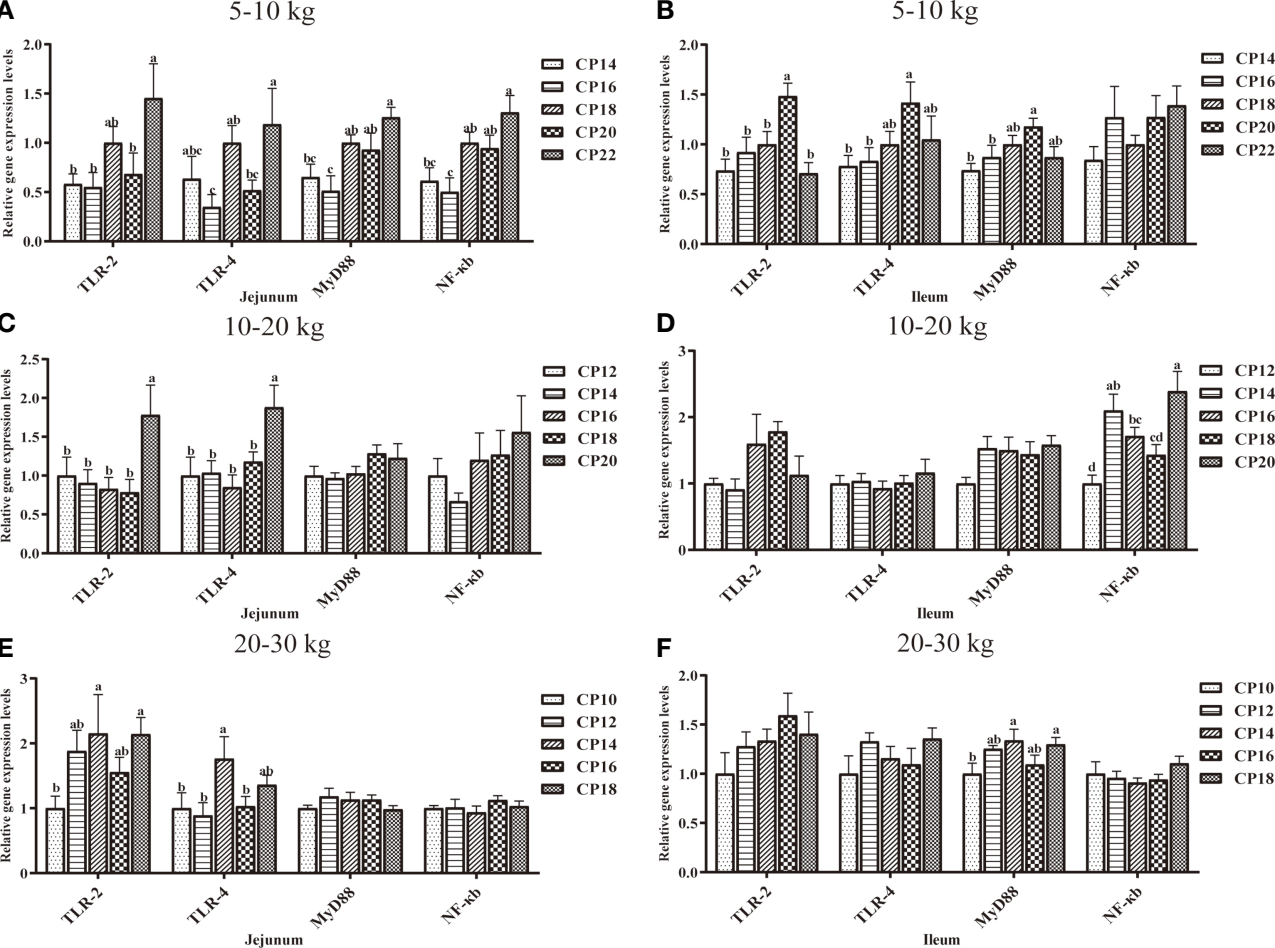
Effects of dietary crude protein (CP) levels on the TLR-MyD88-NF-κB signaling pathway-related gene expression in the small intestinal mucosa of Huanjiang mini-pigs during 5−10 kg (**A**, jejunum; **B**, ileum), 10−20 kg (**C**, jejunum; **D**, ileum), and 20−30 kg (**E**, jejunum; **F**, ileum)t growth stages. Data are presented as means ± SE (*n* = 6−8 per group). Different superscript letters indicate significant differences among the five groups (*P* < 0.05). TLR, toll-like receptors; MyD88, myeloid differentiation factor 88; NF-κB, nuclear factor kappa B.

The effects of dietary CP levels on the expression of immunity-related genes in the small intestinal mucosa of pigs are shown in **Figures 6**, **7**. In the 5−10 kg growth stage (**Figures 6A, B**, **7A, B**), the 18% CP diet downregulated (*P* < 0.05) jejunal *IL-6* expression compared to the 14, 16, and 22% CP groups and *TNF-α* expression compared to the 20% CP group, whereas the 18 and 20% CP diets upregulated (*P* < 0.05) *TNF-α* expression compared to the 14, 16, and 22% CP groups. Additionally, the 22% CP diet upregulated (*P* < 0.05) jejunal *IFN-γ* expression compared to the 14 and 16% CP groups and *IL-10* expression compared to the 16 and 20% CP groups. The 18 and 20% CP diets upregulated (*P* < 0.05) jejunal *MyD88* and *NF-κB* expressions compared to the 16% CP group and in the 22% CP group compared to the 14 and 16% CP groups. In addition, the 18% CP diet upregulated (*P* < 0.05) jejunal *TLR-4* expression compared to the 16% CP group, and the 22% CP diet upregulated (*P* < 0.05) the *TLR-2* expression compared to the 14, 16, and 20% CP groups and *TLR-4* expression compared to the 16 and 20% CP groups. Moreover, the 20% CP diet upregulated (*P* < 0.05) ileal *IFN-γ* expression compared to the 14% CP group and *TLR-4* and *MyD88* expressions compared to the 14 and 16% CP groups. *IL-4* and *TLR-2* expressions were also upregulated (*P* < 0.05) compared to the other CP groups.

In the 10−20 kg growth stage (**Figures 6C, D**, **7C, D**), the 16% CP diet downregulated (*P* < 0.05) jejunal *IL-6* expression compared to the 12% CP group, while the 20% CP diet upregulated (*P* < 0.05) *IL-6* expression compared to the 16 and 18% CP groups. *TNF-α*, *IFN-γ*, *TLR-2*, and *TLR-4* expressions were upregulated (*P* < 0.05) compared to the other four CP groups. In the ileum, the 14−18% CP diets upregulated (*P* < 0.05) *IL-1β* expression compared to the 12% CP group, and the 16% diet upregulated (*P* < 0.05) *IL-4* expression compared to the 12 and 14% CP groups. The 18% CP diet downregulated (*P* < 0.05) *NF-κB* expression compared to the 14% CP group, whereas the 20% CP diet upregulated (*P* < 0.05) *NF-κB* expression compared to the 12, 16, and 18% CP groups.

Additionally, in the 20−30 kg growth stage (**Figures 6E, F**, **7E, F**), the 12−16% CP diets upregulated (*P* < 0.05) jejunal *TLR-2* expression, as well as jejunal *IL-10* expression in the 14 and 18% CP groups, compared to the 10% CP group. The 14% CP diet upregulated (*P* < 0.05) jejunal *TLR-4* expression compared to the 10, 12, and 16% CP groups. In the ileum, the 12 and 14% CP diets upregulated (*P* < 0.05) *IL-4* expression compared to the 10% CP group, and the 14 and 18% CP diets upregulated (*P* < 0.05) *IL-6* expression compared to the 10% CP group.

### Tight Junction Protein-Related Gene Expression in the Small Intestinal Mucosa of Huanjiang Mini-Pigs

**Figure 8:**
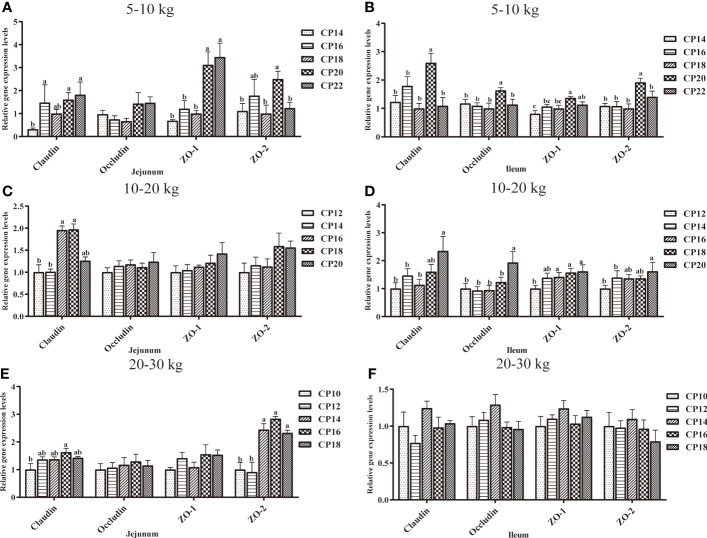
Effects of dietary crude protein (CP) levels on the tight junction protein-related gene expression in the small intestinal mucosa of Huanjiang mini-pigs during 5−10 kg (**A**, jejunum; **B**, ileum), 10−20 kg (**C**, jejunum; **D**, ileum), and 20−30 kg (**E**, jejunum; **F**, ileum) growth stages. Data are presented as means ± SE (*n* = 6−8 per group). Different superscript letters indicate significant differences among the five groups (*P* < 0.05). ZO, zonula occludens.

The effects of dietary CP levels on the expression of tight junction protein-related genes in the small intestinal mucosa of pigs are shown in **Figure 8**. In the 5−10 kg growth stage (**Figures 8A, B**), the 16, 20, and 22% CP diets upregulated (*P* < 0.05) jejunal *Claudin* expression compared to the 14% CP group, the 20 and 22% CP diets upregulated (*P* < 0.05) jejunal *ZO-1* expression compared to the 14−18% CP groups, and the 20% CP diet upregulated (*P* < 0.05) jejunal *ZO-2* expression compared to the 14, 18, and 22% CP groups. In the ileum, the 20% CP diet upregulated (*P* < 0.05) *ZO-1* expression compared to the 14−18% CP groups; *ZO-2*, *Occludin*, and *Claudin* expressions compared to the other four CP groups; and *ZO-1* expression in the 22% CP group compared to the 14% CP group. In the 10−20 kg growth stage (**Figures 8C, D**), the 16 and 18% CP diets upregulated (*P* < 0.05) jejunal *Claudin* expression compared to the 12 and 14% CP groups. In the ileum, the 16−20% CP diets upregulated (*P* < 0.05) *ZO-1* expression compared to the 12% CP group, whereas the 20% CP diet upregulated (*P* < 0.05) *ZO-2* expression compared to the 12 and 14% CP groups. Moreover, the 20% diet upregulated (*P* < 0.05) the *Claudin* expression compared to the 12−16% CP groups and *Occludin* expression compared to the other four CP groups. In the 20−30 kg growth stage (**Figures 8E, F**), the 16% CP diet upregulated (*P* < 0.05) jejunal *Occludin* expression compared to the 10% CP group, whereas the 14–18% CP diets upregulated (*P* < 0.05) *ZO-2* expression compared to the 10 and 12% CP groups. However, there were no significant differences (*P* > 0.05) in the expression of tight junction protein-related genes in the ileum of pigs in the five CP groups.

### Mucin-Related Gene Expression in the Small Intestinal Mucosa of Huanjiang Mini-Pigs

**Figure 9:**
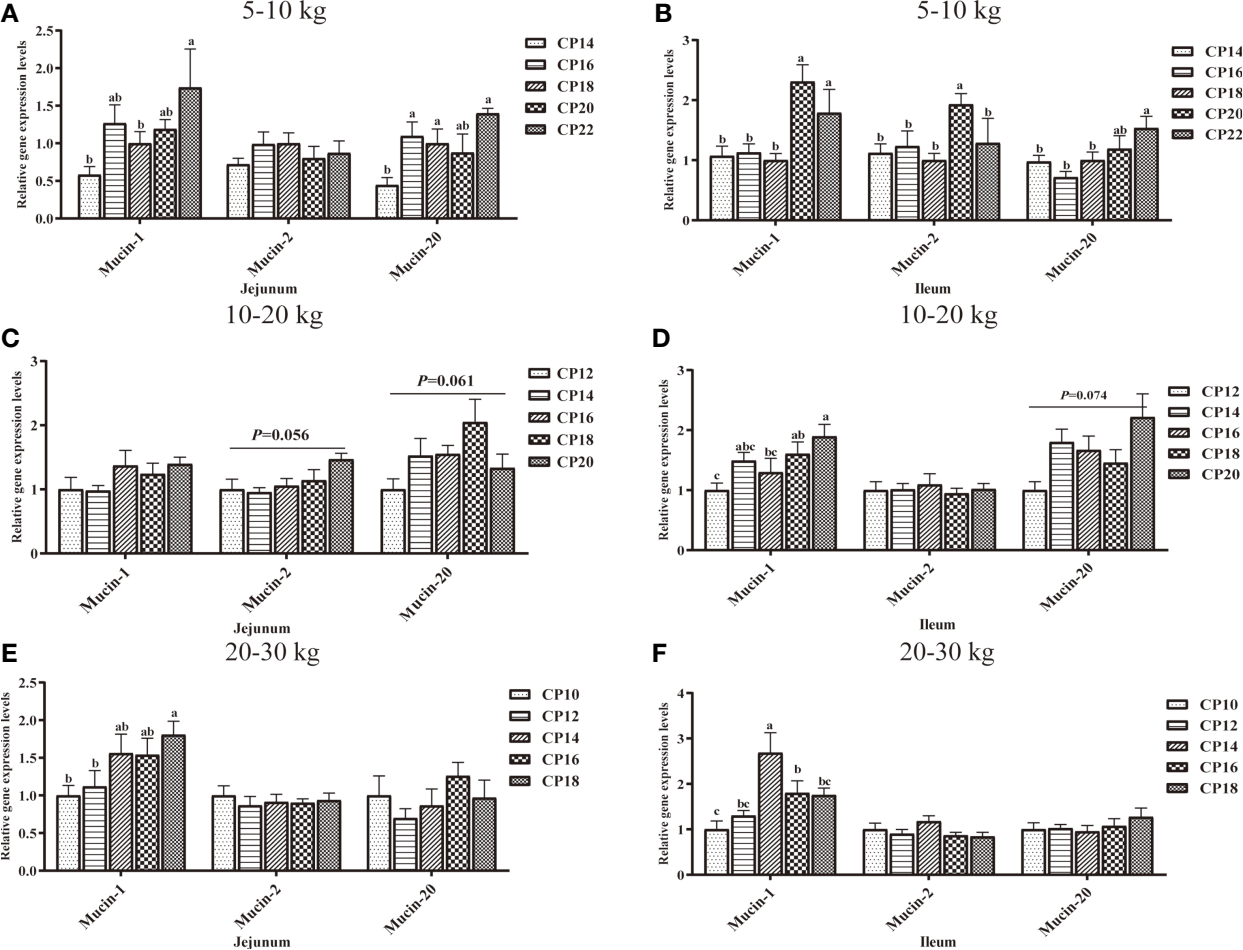
Effects of dietary crude protein (CP) levels on the mucin-related gene expression in the small intestinal mucosa of Huanjiang mini-pigs during 5−10 kg (**A**, jejunum; **B**, ileum), 10−20 kg (**C**, jejunum; **D**, ileum), and 20−30 kg (**E**, jejunum; **F**, ileum) growth stages. Data are presented as means ± SE (*n* = 6−8 per group). Different superscript letters indicate significant differences among the five groups (*P* < 0.05).

The effects of dietary CP levels on mucin-related gene expression in the small intestinal mucosa of pigs during different growth stages are shown in **Figure 9**. In the 5−10 kg growth stage (**Figures 9A, B**), the 16, 18, and 22% CP diets upregulated (*P* < 0.05) jejunal *Mucin-20* expression compared to the 14% CP group, the 20% CP diet upregulated (*P* < 0.05) ileal *Mucin-1* expression compared to the 14−18% CP groups and *Mucin-2* expression compared to the other four CP groups. Moreover, the 22% CP diet upregulated (*P* < 0.05) jejunal *Mucin-1* expressions compared to the 14 and 18% CP groups and ileal *Mucin-1* and *Mucin-20* expressions compared to the 14−18% CP groups. In the 10−20 kg growth stage (**Figures 9C, D**), the 18% CP diet displayed an upregulated tendency (*P* = 0.061) for jejunal *Mucin-20* expression compared to the other four CP groups, an upregulated tendency (*P* = 0.056) for jejunal *Mucin-2* expression in the 20% CP group compared to the 12−16% CP groups and an upregulated tendency (*P* = 0.074) for ileal *Mucin-20* expression in the 20% CP group compared to the 12% CP group. In addition, the 18 and 20% CP diets upregulated (*P* < 0.05) ileal *Mucin-1* expression compared to the 12% CP group. In the 20−30 kg growth stage (**Figures 9E, F**), the 14% CP diet upregulated (*P* < 0.05) ileal *Mucin-1* expression compared to the other four CP groups, while the 16% CP diet upregulated *Mucin-1* expression compared to the 10% CP group. Moreover, the 18% CP diet upregulated (*P* < 0.05) jejunal *Mucin-1* expression compared to the 10 and 12% CP groups.

## Discussion

Dietary nutrient requirements for pigs, especially CP levels, vary according to genotype, physiological status, environmental conditions, and other factors. Moreover, the intestinal mucosal barrier not only participates in nutrient absorption but also serves as the first line of defense in immunity against external pathogens (18). Damage to the intestinal mucosal barrier can be caused by pathogenic invasion, which induces intestinal inflammation and diarrhea in piglets (19). As a major macronutrient, dietary CP is essential for pig growth, development, and health. Therefore, the present study evaluated the effects of dietary CP levels on diarrhea incidence, immunity, and intestinal barrier function in Huanjiang mini-pigs. The results showed that higher CP levels were associated with higher diarrhea incidence, intestinal inflammation, and impaired intestinal barrier function, and the optimal dietary CP levels were 16, 14, and 12% for the 5−10, 10−20, and 20−30 kg growth stages of Huanjiang mini-pigs, respectively.

Diarrhea in piglets is common in both neonatal and post-weaning stages, causes a high level of mortality, and can seriously affect the outcome of extensive animal production (20). Therefore, optimization of dietary CP levels has been suggested as a crucial nutritional strategy for reducing the incidence of diarrhea in swine production. Previous studies have shown that feeding diets containing lower levels of dietary CP is the most efficient way to reduce diarrhea incidence in weaned pigs (19, 21). Consistent with previous studies, our results showed a reduction in the incidence of diarrhea in the lower-level CP groups at different growth stages. Several studies have reported that piglets receiving a low-CP diet supplemented with amino acids showed growth performance comparable to that of piglets fed with a higher CP diet (22, 23). However, our previous study revealed that lower levels of CP in the diet had no impact on growth performance in the early stages (5−10 and 10−20 kg growth stages) of pig growth, whereas the 14−16% CP diets improved the ADFI of pigs compared to the 18% CP diet at the 20−30 kg growth stage (9). Consistent with our findings, a recent study also indicated that reducing dietary CP levels from 22 to 16% could decrease diarrhea incidence in the early growth stages of pig without affecting growth performance (24). Therefore, these findings indicate that lower CP levels can reduce the incidence of diarrhea during different growth stages in Huanjiang mini-pigs. However, the 22% CP diet decreased the diarrhea index compared to the 20% CP group, while the degree of diarrhea did not change, which may be related to the total number of diarrhea incidences in the 22% CP group. Therefore, the final diarrhea rate may have been decreased because of the calculation formula. Further studies are required to clarify this inconsistency.

Small intestinal morphology indices, such as VH, CD, and VH to CD ratio, are generally used as indicators of piglet intestinal absorption and digestion ability (25). Research has shown that a diet consisting of 26% CP decreased the VH to CD ratio and ileal VH in weaned piglets compared to the 18% CP diet (8). In another study, dietary reduction of dietary CP levels from 16 to 10% significantly decreased the intestinal VH to CD ratio (26). In the present study, there were no significant differences in the VH, CD, and VH to CD ratios during the different growth stages, except for a significant increase in jejunal VH (in the 18% CP group) at the 5−10 kg growth stage. These inconsistencies might be related to different pig breeds; however, further studies are needed to determine the exact reason.

Cytokines are potential modulating indicators of the inflammatory responses of a host. Pro-inflammatory cytokines, such as IL-1β, IL-6, IL-8, TNF-α, and IFN-γ, can regulate the inflammatory response, and their levels reflect the inflammatory status of the host body (27). In the present study, a higher-level CP diet (22%) increased the plasma IL-1β, IL-6, IL-8, and TNF-α levels compared to the 14−20% CP diets at the 5−10 kg growth stage. These results were not consistent in the later stages, as the plasma IL-1β level in the 20% CP group was lower than that in the 14% CP group, as was the TNF-α level in the 18−20% CP groups compared to the 12% CP group at the 10−20 kg growth stage. These findings were consistent with results from a previous study that reported that a higher CP diet (22.5%) could increase serum IL-β and TNF-α levels compared to a lower CP diet (17.6%) and cause mild inflammation (28). Anti-inflammatory cytokines such as IL-10 and TGF-β are involved in suppressing inflammation. In the present study, decreased levels of IL-10 and TGF-β were observed in the 22% CP group at the 5−10 kg growth stage, and TGF-β and IL-10 levels were decreased in the 16 and 18% CP groups, respectively, at the 20−30 kg growth stage, which is in agreement with previous findings (29).

Further investigation of intestinal cytokines revealed that jejunal and ileal IL-10 levels were increased in the 20% CP group compared to the 14% CP group at the 5−10 kg growth stage, as well as in the 16% CP group compared to the 12% CP group at the 10−20 kg growth stage. Only ileal IL-10 was increased in the 16% CP group compared to the other CP groups. These findings are in agreement with our previous findings, which indicated that dietary CP levels at 18.42, 16.70, and 14.00% were optimal for Huanjiang mini-pigs at the 5−10, 10−20, and 20−30 kg growth stages, respectively (9). In addition, our results also showed that the 18% CP diet at the 5−10 kg growth stage and the 16% CP diet at the 10−20 kg growth stage downregulated jejunal *IL-6* expression, whereas higher-level CP diets upregulated *IFN-γ* and *TNF-α* expressions. However, the upregulation of anti-inflammatory gene expression was not consistent during different growth stages. Therefore, it is postulated that lower CP diets could enhance the immune system, which does not require the expression of other anti-inflammatory genes (30). This may also be the reason for the observed downregulation of *Mucin-2* and *Mucin-20* expressions in the jejunum and ileum at the 5−10 kg growth stage, as mucins are important for local defense against enteric pathogens (31, 32). A recent study also reported that a lower dietary CP level (16%) could reduce mucin secretion and inflammation in piglets (29). A possible mechanism might be that a lower CP diet may restrict protein synthesis for cell turnover due to the limiting amino acids.

Tight junction proteins, including ZO-1, ZO-2, Occludin, and Claudin are the most important components of the intestinal epithelial barrier. A layer of epithelial cells forms the intestinal barrier, joined together by tight junction protein expression (21, 33). Previous studies have shown that the upregulation of pro-inflammatory cytokines can induce the alteration of tight junction proteins, which are associated with the downregulation of *ZO-1*, *Occludin*, and *Claudin* expressions (34). In the present study, jejunal expression of *Claudin* and *ZO-1* was upregulated in the 20 and 22% CP groups, and jejunal *ZO-2*, ileal *Claudin*, *Occludin*, *ZO-1*, and *ZO-2* were upregulated in the 20% CP group at the 5−10 kg growth stage. In addition, tight junction proteins were also upregulated in the 16 and 18% CP groups at the 10−20 kg growth stage, and jejunal *Claudin* and *ZO-2* were upregulated in the 16% and 14−18% CP groups, respectively, at the 20−30 kg growth stage. These findings are contrary to those reported by Wu et al. (21), and the different dietary CP levels and pig breeds could explain this difference.

Several studies have reported that higher-level CP diets (CP > 20%) can increase pro-inflammatory factors and upregulate the expression levels of intestinal *TLR-4*, *MyD88*, and *NF-κB* in piglets, which can lead to decreased immune function (35). Similarly, our results showed that the 22% CP diet upregulated the jejunal expression levels of *TLR-2*, *TLR-4*, *MyD88*, and *NF-κB*, whereas the 20% CP diet upregulated ileal *TLR-2*, *TLR-4* and *MyD88* at the 5−10 kg growth stage. Moreover, a higher dietary CP level (20%) was also found to upregulate jejunal *TLR-2*, *TLR-4* and ileal *NF-κB* expression at the 10−20 kg growth stage. NF-κB is a nuclear transcription factor, which regulates the expression of various genes involved in inflammation. Moreover, TLRs and MyD88 are upstream regulatory factors of NF-κB (36, 37). Therefore, these findings indicate that the upregulation of the TLR-MyD88-NF-κB signaling pathway during different growth stages, in agreement with previous data (35, 38), and may be harmful to the mucosal immune responses in the intestine.

## Conclusion

In summary, dietary CP levels > 16% at the 5−10 kg and 10−20 kg growth stages and > 14% at the 20−30 kg growth stage increased the potential risk of diarrhea incidence in Huanjiang mini-pigs. Moreover, higher dietary CP levels also increase intestinal inflammatory status by decreasing intestinal immune functions and may activate the TLR-MyD88-NF-κB signaling pathway to induce intestinal inflammation. Collectively, these findings also suggest optimal dietary CP levels of 16, 14, and 12% for Huanjiang mini-pigs during the 5−10 kg, 10−20 kg, and 20−30 kg growth stages, respectively.

## Data Availability Statement

The data used to support the findings are all included in the article or **Supplementary Material**. Further inquiries can be directed to the corresponding author.

## Ethics Statement

The animal study was reviewed and approved by the Animal Care and Use Committee of the Institute of Subtropical Agriculture, Chinese Academy of Sciences (No. ISA-2019-4-29). Written informed consent was obtained from the owners for the participation of their animals in this study.

## Author Contributions

XK designed the experiment. YL, QZ, and XZ contributed to animal experiments, sample collection, and analysis. YL, MA, and XK were responsible for writing the original draft and manuscript modification. All authors contributed to the article and approved the submitted version.

## Funding

The present study was jointly supported by the Production and Research Talent Support Project of the CAS Wang Kuancheng Initiative Talent Program, and Special Funds for Construction of Innovative Provinces in Hunan Province (2019RS3022).

## Conflict of Interest

The authors declare that the research was conducted in the absence of any commercial or financial relationships that could be construed as a potential conflict of interest.

## Publisher’s Note

All claims expressed in this article are solely those of the authors and do not necessarily represent those of their affiliated organizations, or those of the publisher, the editors and the reviewers. Any product that may be evaluated in this article, or claim that may be made by its manufacturer, is not guaranteed or endorsed by the publisher.
