# Supplementary material for: Dietary Crude Protein Levels Alter Diarrhea Incidence, Immunity, and Intestinal Barrier Function of Huanjiang Mini-Pigs During Different Growth Stages
Source: Front Immunol. 2022 Jul 7;13:908753. doi: 10.3389/fimmu.2022.908753 (PMC9301461; doi:10.3389/fimmu.2022.908753)
Supplement: Supplementary file 1 [file DataSheet_1.docx]

| Items (μm) | Dietary CP levels (%) | | | | |
| --- | --- | --- | --- | --- | --- |
| 5−10 kg growth stage | 14 | 16 | 18 | 20 | 22 |
| Jejunum |  |  |  |  |  |
| Villus height | 327.30±64.62^b^ | 316.17±36.19^b^ | 399.50±64.43^a^ | 320.60±13.34^b^ | 323.60±24.92^b^ |
| Crypt depth | 167.19±12.23 | 172.04±12.69 | 176.22±11.14 | 148.29±10.60 | 148.85±7.35 |
| VH/CD | 2.42±0.22 | 2.10±0.17 | 2.33±0.16 | 2.49±0.09 | 2.33±0.18 |
| Ileum |  |  |  |  |  |
| Villus height | 289.80±25.24 | 304.90±19.97 | 290.30±23.46 | 331.70±54.73 | 292.60±15.95 |
| Crypt depth | 163.34±7.41 | 158.23±8.34 | 159.54±5.78 | 146.03±7.22 | 149.22±6.80 |
| VH/CD | 1.93±0.12 | 2.08±0.09 | 1.93±0.13 | 2.54±0.37 | 2.11±0.17 |
| 10−20 kg growth stage | 12 | 14 | 16 | 18 | 20 |
| Jejunum |  |  |  |  |  |
| Villus height | 402.60±24.66 | 416.15±33.89 | 423.74±25.36 | 399.11±14.04 | 383.50±25.90 |
| Crypt depth | 193.41±15.74 | 172.40±15.14 | 173.88±12.26 | 174.31±7.15 | 165.70±8.40 |
| VH/CD | 2.11±0.12 | 2.44±0.13 | 2.46±0.09 | 2.31±0.09 | 2.33±0.16 |
| Ileum |  |  |  |  |  |
| Villus height | 341.38±25.97 | 344.33±23.22 | 337.83±23.72 | 335.73±6.57 | 355.62±10.53 |
| Crypt depth | 173.44±8.15 | 161.90±6.95 | 173.99±7.87 | 164.51±6.21 | 171.51±11.49 |
| VH/CD | 1.97±0.12 | 2.15±0.17 | 1.93±0.08 | 2.07±0.10 | 2.10±0.10 |
| 20−30 kg growth stage | 10 | 12 | 14 | 16 | 18 |
| Jejunum |  |  |  |  |  |
| Villus height | 433.29±37.10 | 434.04±28.71 | 423.74±25.36 | 399.11±14.04 | 383.50±25.90 |
| Crypt depth | 198.88±14.38 | 162.48±13.30 | 173.88±12.26 | 174.31±7.15 | 165.70±8.40 |
| VH/CD | 2.19±0.13 | 2.76±0.24 | 2.46±0.09 | 2.31±0.09 | 2.33±0.16 |
| Ileum |  |  |  |  |  |
| Villus height | 341.96±24.96 | 347.49±14.62 | 343.58±22.37 | 360.68±31.02 | 330.28±18.56 |
| Crypt depth | 166.12±6.46 | 166.42±4.17 | 159.95±7.35 | 161.62±5.48 | 167.67±9.13 |
| VH/CD | 2.06±0.12 | 2.09±0.08 | 2.15±0.13 | 2.23±0.16 | 1.98±0.10 |
